# PREDICTING MORTALITY IN ADULTS USING TRANSFORMERS: INSIGHTS FROM THE HEALTH AND RETIREMENT STUDY

**DOI:** 10.1093/geroni/igae098.0559

**Published:** 2024-12-31

**Authors:** Jordan Weiss, Alaleh Azhir, Nilam Ram, David Rehkopf

**Affiliations:** Stanford University, Stanford, California, United States; Harvard University, Cambridge, Massachusetts, United States; Stanford University, Stanford, California, United States; Stanford University, Palo Alto, California, United States

## Abstract

We introduce a natural language processing (NLP)-based prediction model to predict next-wave mortality in respondents from the US-based Health and Retirement Study (HRS). Akin to how NLP models can be used to predict future words in a sentence, we use a transformer architecture to build embeddings of a respondent’s demographic, social, behavioral, and health histories which we then use to predict their probability of death at subsequent waves. Our model surpasses performance metrics established by conventional statistical models (average precision score = 0.900 versus 0.395), and provides new avenues for personalized healthcare in an observational setting.

